# *In vivo* gene silencing following non-invasive siRNA delivery into the skin using a novel topical formulation

**DOI:** 10.1016/j.jconrel.2014.10.022

**Published:** 2014-12-28

**Authors:** Vikas Hegde, Robyn P. Hickerson, Sitheswaran Nainamalai, Paul A. Campbell, Frances J.D. Smith, W.H. Irwin McLean, Deena M. Leslie Pedrioli

**Affiliations:** a Centre for Dermatology and Genetic Medicine, Division of Molecular Medicine, University of Dundee, Dundee DD1 5EH, Scotland, UK; b Carnegie Physics Laboratory, University of Dundee, Dundee DD1 4HN, Scotland, UK

**Keywords:** RNAi-based therapeutics, Topical formulation, Transepidermal siRNA delivery, Luciferase reporter mouse model, Filaggrin, siRNA, small interfering RNA, MO, morpholino antisense oligonucleotide, FLG, filaggrin, PG, propylene glycol

## Abstract

Therapeutics based on short interfering RNAs (siRNAs), which act by inhibiting the expression of target transcripts, represent a novel class of potent and highly specific next-generation treatments for human skin diseases. Unfortunately, the intrinsic barrier properties of the skin combined with the large size and negative charge of siRNAs make epidermal delivery of these macromolecules quite challenging. To help evaluate the *in vivo* activity of these therapeutics and refine delivery strategies we generated an innovative reporter mouse model that predominantly expresses firefly luciferase (*luc2p*) in the paw epidermis — the region of murine epidermis that most closely models the tissue architecture of human skin. Combining this animal model with state-of-the-art live animal imaging techniques, we have developed a real-time *in vivo* analysis work-flow that has allowed us to compare and contrast the efficacies of a wide range nucleic acid-based gene silencing reagents in the skin of live animals. While inhibition was achieved with all of the reagents tested, only the commercially available “self-delivery” modified Accell-siRNAs (Dharmacon) produced potent and sustained *in vivo* gene silencing. Together, these findings highlight just how informative reliable reporter mouse models can be when assessing novel therapeutics *in vivo*. Using this work-flow, we developed a novel clinically-relevant topical formulation that facilitates non-invasive epidermal delivery of unmodified and “self-delivery” siRNAs. Remarkably, a sustained > 40% *luc2p* inhibition was observed after two 1-hour treatments with Accell-siRNAs in our topical formulation. Importantly, our ability to successfully deliver siRNA molecules topically brings these novel RNAi-based therapeutics one-step closer to clinical use.

## Graphical abstract

Unique epidermal mice were combined with IVIS live animal imaging to define nucleic acid-based therapeutic afficacies *in vivo* and develop a novel clinic-ready siRNA topical delivery formulation.

**Figure:**
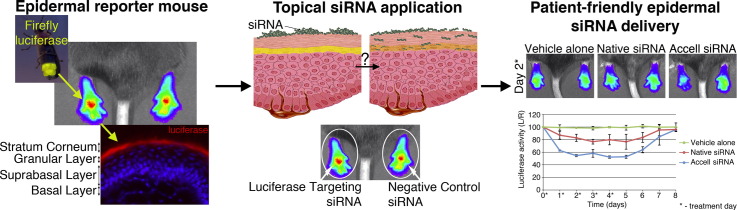

## 1 Introduction

Diagnosis, management and/or treatment of human skin conditions represent a significant healthcare burden. In the UK, 20–30% of the population has been diagnosed with a skin disease, ~ 15% of all general practitioner consultations involve a skin condition, and dermatological prescriptions are second only to those for painkillers [1]. Advances in molecular genetics and completion of the human genome project have significantly improved many aspects of clinical dermatology. The identification of pathogenic mutations has greatly improved rare heritable skin disorder diagnoses [2,3] and the sub-classification of common skin disorders like ichthyosis vulgaris and atopic dermatitis eczema [4,5]. Despite these advances, relatively little progress has been made towards developing specific and effective treatments for human skin diseases.

Nucleic acid-based therapeutics, including RNA interference (RNAi) therapies that function *via* small interfering RNAs (siRNAs), have emerged as a promising new class of highly specific, disease-targeted treatments for a wide range of human diseases [6,7]. The skin is the most accessible organ in the body, and therefore, represents a strong candidate for siRNA therapies [8]. Indeed, these therapeutics have yielded promising preclinical data for a number of skin conditions, including psoriasis [9], allergic skin disease [10–12], epidermolysis bullosa simplex [13], epidermolytic palmoplantar keratoderma [14] and pachyonychia congenita [15,16]. The first siRNA to specifically target a mutant allele was evaluated by intradermal injection in a phase 1b trial for pachyonychia congenita [17]. Unfortunately, this method of delivery cannot be pursued for future pachyonychia congenita treatments due to the intense pain associated with injection. A second, intradermal injection based phase 1 trial, using a self-delivering RNAi compound (sd-rxRNA from RXi Pharmaceuticals, Westborough, MA) designed to reduce scarring following planned surgery, was recently completed (RXI-109, http://www.rxipharma.com). Although these studies have reported encouraging results, the technical bottleneck of efficiently overcoming the skin's barrier properties in a patient-friendly and clinically-relevant manner has slowed translation towards clinical use [17].

Pain-free, non-invasive topical application of siRNA formulations that facilitate siRNA delivery to the disease-relevant layers of the epidermis would provide the ideal treatment platform for human skin conditions. Unfortunately, because of the large size (molecular weight ≈ 13.5 kDa) and negative charge of siRNA molecules, both of which impede *stratum corneum* (the outermost barrier of the skin) and cell membrane penetration, topical delivery strategies have only been moderately successful [12,18,19]. siRNAs have been delivered into mouse skin using minimally invasive techniques such as electroporation [20], iontophoresis [21] and coated steel and dissolvable microneedles [22,23]. However, these delivery strategies have not yet progressed to the clinical trial phase. In the event that siRNAs are able to successfully penetrate the *stratum corneum*, siRNA-mediated gene regulation still requires keratinocyte uptake. “Self-delivery” siRNAs, like commercially available Accell™-siRNA, which carry chemical modifications that enhance cellular uptake, have been developed and effectively inhibit gene expression without the use of transfection reagents *in vitro* and *in vivo* [24–28].

Evaluation and refinement of *in vivo* delivery approaches have been hampered by the lack of reliable animal models. Green fluorescent protein (GFP) reporter mouse models have provided beneficial insights into epithelial appendage biology and identified potential drug targets for the treatment of hair loss, as well as other skin and hair disorders [29,30]. siRNA potency [31] and delivery using microneedles [23] have been evaluated *in vivo* by monitoring fluorescence in a dual reporter mouse model. Previous studies have, however, demonstrated that luciferase imaging is more sensitive than fluorescence imaging [32]. We, therefore, developed a unique transgenic reporter mouse model, where luciferase expression is confined to the epidermis, for validating new dermatological therapeutics and refining drug delivery to skin. Here, we used this innovative animal model to compare and contrast the real-time *in vivo* efficacies of unmodified or modified siRNAs, morpholino antisense oligonucleotides and *in vivo* transfection reagents in live animals. Excitingly, using this real-time live animal imaging work-flow, we have developed a relatively simple “gene cream” that facilitates epidermal delivery of both unmodified and “self-delivery”-modified siRNAs.

## 2 Materials & methods

### 2.1 Generation of *FLG*-*luc2p* bioluminescence reporter mice

A 10 kb human filaggrin promoter fragment was derived from a bacterial artificial chromosome (BAC clone RP1-14N1) *via* a two-step recombineering process. A 10.1 kb fragment containing a 5′ *Xho*I restriction site, ~ 10 kb upstream of the transcription start site, exon 1 (partial 5′UTR), the first 18-bp of intron 1, and a 3′ *Mlu*I restriction site was amplified from the BAC clone using primers mentioned in Supplementary Table 1. A second, 483-bp fragment containing a 5′ *Mlu*I restriction site, the last 459-bp of intron 1, the start of exon 2 encompassing the remainder of the 5′UTR and a 3′ *Hind*III restriction site was amplified from the same BAC clone. Fragments were sequence-verified and ligated *via* their *Mlu*I sites, generating the 10.6 kb hFLG-10k human filaggrin promoter fragment. Finally, the *FLG*-10k promoter driven mammalian codon-optimized, protein destabilized firefly luciferase gene (*luc2p*) transgene (*FLG*-10k-*luc2p*) was generated by cloning the *FLG*-10k fragment into pGL4.21 *via* the *Xho*I and *Hind*III restriction sites. Following sequence and expression verification, the *FLG*-10k-*luc2p* construct was used to generate a single-copy transgenic C57BL/6J mouse *via* embryonic stem cell gene targeting into the murine *Rosa26* locus (TaconicArtemis GmbH, Cologne, Germany).

### 2.2 *In vivo* live-animal imaging

*FLG*-*luc2p*^+/−^ and *FLG*-*luc2p*^−/−^ (WT; wild type) mice (6–12 weeks old) were housed and used for these studies following the husbandry and experimental guidelines defined by the UK animal welfare act. *In vivo* live-animal imaging of *FLG-luc2p*^+/−^ mice was performed as previously described [14], using the Xenogen IVIS 200 imaging system (PerkinElmer, Waltham, MA). Unless otherwise stated, all images were captured with 1 second (s) exposures. The resulting light emission was quantified using LivingImage software 3.0 (PerkinElmer). %L/R ratios were calculated by dividing left paw luciferase light emission (LLE) by right paw LLE. Baseline luciferase bioluminescent activity was defined for each experimental animal by monitoring hindpaw luciferase activity at 24 hour (h) intervals for 5–6 days prior to treatment.

### 2.3 Tissue collection for qRT-PCR and Western blot analysis

*FLG*-*luc2p*^+/−^ (n = 10) and WT (n = 5) mice were imaged using the IVIS 200. Fur was shaved prior to imaging abdominal dorsal and ventral regions. Following imaging, animals were sacrificed. Tissues were harvested, divided in half, then snap-frozen in liquid nitrogen and stored at − 80 °C.

For total RNA extraction, tissues were lysed using TissueLyser LT system (Qiagen, Crawley, UK) in 700 μl of buffer RLT containing 1% (v/v) β-mercaptoethanol for 2 minutes (min) at 4 °C at 50 Hz and further homogenized by passing through a RNeasy-QIAshredder column (Qiagen). Total RNA was isolated using the RNeasy Mini kit (Qiagen) according to the manufacturer's instructions and stored at − 80 °C. Total RNA was reverse transcribed using the High Capacity cDNA Reverse Transcription kit (Life Technologies, Paisley, UK) according to the manufacturer's instructions and stored at − 20 °C.

Whole tissue lysates were prepared by manual grinding tissues in liquid nitrogen with a mortar and pestle and reconstituted in 100–150 μl of RIPA buffer [33] containing 1% (v/v) protease inhibitor cocktail (Sigma-Aldrich, UK). Lysates were incubated for 15 min on ice, cleared *via* centrifugation (16,000 ×*g*) for 20 min at 4 °C and the supernatant was stored at − 80 °C.

### 2.4 Taqman quantitative real-time PCR (qRT-PCR)

*Luc2p* mRNA expression levels were analyzed in triplicate by qRT-PCR using a custom firefly luciferase *luc2p* TaqMan assay: probe (5′ ACAACCAGCGCCATTC 3′) and primers (forward — 5′ GGCTACGGCCTGACAGAA 3′ reverse — 5′ CTGCGCCAGGCTTGTC 3′) in Type-it™ Fast SNP PCR Master Mix (Qiagen) using a 7900HT Fast Real-Time PCR System (Life Technologies). 25 ng template cDNA was used for each reaction and *relative* abundances were calculated by ΔΔC*_T_* method using GAPDH as the reference gene. Comparative threshold cycle (*C_T_*) values were calculated using SDS 2.2 software (Life Technologies).

### 2.5 Immunoblotting

5 μg of whole tissue lysates were resolved in 4–12% Bis–Tris denaturing NuPAGE gels (Life Technologies) and transferred to nitrocellulose membranes. Membranes were cut at the 50 kDa marker, blocked for 1 h at RT in blocking buffer (3% BSA; 1×TBS, 0.5% Tween 20), and incubated with primary antibodies diluted in blocking buffer overnight at 4 °C. The top half of the membrane was probed with a rabbit polyclonal antibody to firefly luciferase (GTX125849, Genetex, Irvine, USA) diluted 1:1000 and the bottom half was probed with mouse monoclonal antibody to β-actin (A5441, Sigma-Aldrich, UK) diluted 1:5000. Membranes were washed, incubated with 1:5000 Alexa Fluor® 680 goat anti-rabbit IgG and Alexa Fluor® 680 goat anti-mouse IgG (Life Technologies) and imaged using the LI-COR Odyssey (LI-COR, Cambridge, UK) as previously described [14].

### 2.6 Histology and immunofluorescence

FLG-*luc2p*^+/−^ and WT hindpaw skin tissues were dissected and fixed in neutral buffered formalin for 48 h. Surgical surplus adult human skin samples were acquired *via* the Tayside Tissue Bank, Dundee, UK under ethics approval number TR000281. Tissues were dehydrated, paraffin embedded, sectioned (8 μm) and mounted on superfrost-plus slides (VWR International). For histopathological analyses, sections were deparaffinized and hematoxylin/eosin stained according to standard protocols.

For immunofluoresence staining, 8 μm fresh-frozen hindpaw skin tissues, mounted on superfrost-plus slides, were used. Sections were fixed in cold methanol:acetone (1:1), blocked in 10% (v/v) goat serum/PBS for 15 min at room temperature, and incubated with 1:500 rabbit anti-K1 (ab15580, Abcam, Cambridge, UK), 1:200 rabbit anti-FLG [34] or 1:2500 rabbit anti-Luc (ab21176, Abcam) primary antibodies diluted in 10% (v/v) goat serum/PBS and incubated overnight at 4 °C. Sections were washed with 10% (v/v) goat serum/PBS, probed with 1:350 Alexa Fluor® 568 goat anti-rabbit (Life Technologies) or 1:350 Alexa Fluor® 488 goat anti-rabbit (Life Technologies) for 1 h at room temperature, washed and counterstained with DAPI (1 μg/mL in PBS) for 3 min. Immunofluorescence was imaged using the Eclipse E600 fluorescent microscope (Nikon, UK). Histology was imaged using an Axioskop (Zeiss, USA).

### 2.7 siRNA and MO reagents

Standard, de-salted unmodified or native *Luc2p*-targeting (siLUC2P-2; sense 5′-CGACAAGCCUGGCGCAGUATT-3′), non-targeting control (NCS4; sense 5′-UAGCGACUAAACACAUCAATT-3′) and non-pathway targeting control (siNPT; sense 5′-GCAAGCTGCTGGGGGGCGATT-3′) siRNAs were synthesized by Eurofins MWG Operon (Ebersberg, Germany). Self-delivery modified Accell™-siRNAs were synthesized by Dharmacon Research (Thermo Fisher Scientific). Standard control oligo (5′-CCTCTTACCTCAGTTACAATTTATA-3′) and *luc2p*-targeting LUC2P-2 (5′-TCCATGGTGGCTTTACCAACAGTAC-3′) native and self-delivery modified (Vivo) morpholino antisense oligonucleotides were synthesized by Gene Tools (Philomath, OR, USA).

### 2.8 *In vitro* luciferase assays

Native- and Accell™-modified siLUC2P-2, NSC4 and siNPT siRNAs were evaluated *in vitro* using a stable HaCaT human keratinocyte cell line expressing *luc2p* (pK6a-*luc2p*) (Zhao, 2011 #586). Briefly, 4000 cells/well were plated in 96-well plates in DMEM/10% (v/v) FCS, incubated for 18 h, transferred to DMEM and incubated for a further 2 h. Native- and Accell™-siRNAs were added at the indicated concentrations (n = 8/condition), incubated for 24 h, supplemented with FCS to 2% (v/v) and incubated for an additional 24 h. Cell viability was determined by addition of 2.3 mM (final) resazurin dye (Sigma) and incubation at 37 °C for 90 min. Fluorescence/well was quantified using a micro-titer plate reader (530 nm excitation/590 nm emission). Luciferase activity was assayed using the LUMIstar OPTIMA plate reader (BMG Labtech, Aylesbury, UK) after adding an equal volume of 2 × Luciferin buffer (50 mM Tris/phosphate, pH 7.8, 16 mM MgCl_2_, 2 mM DTT, 2% w/v Triton X-100, 30% w/v Glycerol, 1 mM ATP, 1% w/v BSA, 8 μM sodium pyrophosphate, 0.2 mg/mL Luciferin) to each well. Three biological replicate experiments were performed.

### 2.9 Intradermal paw injections

7.5 μM nucleic-acid solutions containing native- or Accell™-siRNAs (siLUC2P-2 or NSC4) or native- or Vivo-MOs (LUC2P-MO or Control-MO) were prepared in sterile PBS. For *in vivo* transfection reagent studies, 1.5 μM native-siRNA solutions were prepared in sterile PBS. Invivofectamine® 2.0 (Life Technologies) and Injectin (BioCellChallenge, France) 1.5 μM siRNA solutions were prepared according to the manufacturer's instructions.

For each treatment group (3–6 animals/group, as indicated), baseline (i.e. pre-treatment) %L/R luciferase activity ratios were defined using IVIS 200 for 5 consecutive days. Following imaging, left paws received *luc2p*-targeting siRNAs or MOs and right paws received control siRNAs or MOs. For each, a total volume of 40 μL was intradermally injected with a 29-gauge needle. For all control animal cohort injections, 40 μL of vehicle controls were injected into both the left and right paws. Luciferase activity was monitored as described above at 24 hour intervals until %L/R ratio returned to baseline.

### 2.10 Topical siRNA formulation and *in vivo* application

Aquaphor®-siRNA mixtures were prepared by manually mixing 37 μg Aquaphor® healing ointment (Eucerin®, USA) with 300 pmol of native or Accell™-modified siLUC2P-2, NSC4 or siNPT siRNAs using a heat-sealed Pasteur pipette. Aquaphor®-PG-siRNA mixtures were prepared by mixing Aquaphor® healing ointment (29 μg) with PG (Thermo Fisher Scientific) to a final concentration of 20% (v/w) (8 μL) and 300 pmol of native or Accell™-modified siLUC2P-2, NSC4 or siNPT siRNAs. FLG-*luc2p*^+/−^ baseline luciferase activity was defined prior to initial topical formulation application. Following formulation application, the animals were kept anesthetized to allow siRNA penetration (~ 45 min). Each application was repeated for five consecutive days and luciferase activity was measured at 24 hour intervals.

## 3 Results

### 3.1 Generation and characterization of *FLG-luc2p*^+/−^ mouse model

**Fig. 1:**
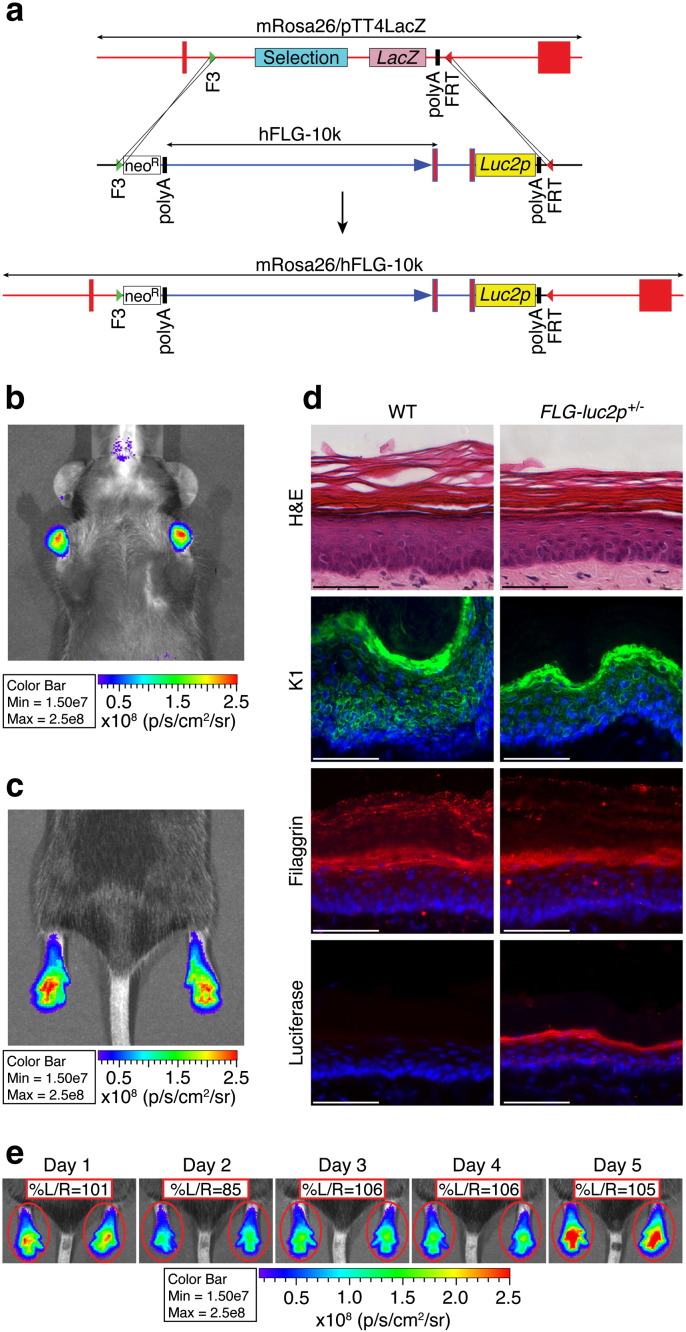
*FLG-luc2p* mouse model. (a) Schematic diagram of the hFLG-10K-*luc2p/Rosa26* knockout-replacement strategy used to generate the *FLG-luc2p* mouse model. The hFLG-10K-*luc2p* transgene contains a 10.6 kb human filaggrin promoter construct fused to the mammalian codon-optimized firefly luciferase gene *luc2p*. Single-copy C57BL/6J *FLG-luc2p* mice were generated via embryonic stem cell gene targeting into the murine *Rosa26*. (b & c) Luciferase expression patterns in *FLG-luc2p*^+/−^ mice were defined using *in vivo* bioluminescent imaging. Signals were strongest in the forepaws and hindpaws, although expression was detected in all skin samples monitored (see Supplementary Fig. 1). (d) WT (*FLG-luc2p*^-/-^) and *FLG-luc2p*^+/−^ hindpaw tissues were hematoxylin/eosin (H&E) stained, or probed with α-keratin 1, α-filaggrin, or α-firefly luciferase antibodies and processed for immunofluorescence microscopy. *FLG-luc2p* reporter gene expression did not effect epidermal architecture (H&E) or alter endogenous K1 expression. Importantly, expression of luciferase and mouse filaggrin in the *stratum granulosum* confirmed that the hFLG-10K-*luc2p* transgene was appropriately expressed in the skin of *FLG-luc2p*^+/−^ mice. Scale bar = 50 µm. (e) *In vivo* imaging of *FLG-luc2p*^+/−^ mice (n = 12) at 24 hour intervals for 5 consecutive days revealed symmetric bioluminescent activity (%L/R ratio ≈ 101 ± 9) in the right and left paws at each time point. Color bar depicts luciferase light emission (LLE) intensity (photons/s/cm^2^/sr) all throughout. %L/R ratios were calculated throughout as follows: (left LLE/right LLE) × 100.

A bioluminescent reporter mouse model was constructed based on the ubiquitous and exclusive expression of filaggrin in the keratohyalin granules of the *stratum granulosum* [35]. This was done using a human filaggrin (*FLG*) promoter driven firefly luciferase transgene (*FLG*-10k-*luc2p*), which contains a 10.6 kb modified *FLG* promoter (~ 10 kb upstream of the *FLG* transcription start site, a fragment of intron 1 and the 5′UTR) and the mammalian codon-optimized, protein destabilized *luc2p* firefly luciferase gene (Fig. 1a). A single-copy *FLG*-*luc2p* transgenic mouse was generated *via* embryonic stem cell gene targeting into the *Rosa26* locus.

*In vivo* live-animal imaging, following luciferin administration, was used to characterize *luc2p* gene expression patterns in *FLG*-*luc2p*^+/−^ mice and revealed strong, bilaterally symmetric bioluminescent activity in the forepaws and hindpaws of these animals (Fig. 1b & c). Much weaker luciferase activity was observed in the tail, ear, snout, perioral and perianal regions, as well as shaved dorsal and ventral skin (Supplementary Fig. 1a–b). Immunoblot and qRT-PCR analysis of skin tissues confirmed that *luc2p* mRNA was most abundant in the hindpaw and that luciferase protein was only detectable by immunoblot in the hindpaw (Supplementary Fig. 1c–d). Finally, immunofluorescence staining demonstrated that both endogenous mouse filaggrin and luciferase were appropriately localized within the *stratum granulosum* of *FLG*-*luc2p*^+/−^ tissues (Fig. 1d).

Histological comparison of fur-covered mouse epidermal, normal human epidermal and mouse paw epidermal tissues (Supplementary Fig. 1e), revealed that the paw epidermis of the mouse is the one region of murine epidermis that most closely models the tissue architecture of human skin. This, together with the strong, bilaterally symmetric, epidermal expression of *luc2p* in the paws of *FLG*-*luc2p*^+/−^ mice (Fig. 1e) suggested that this animal model was well suited for evaluating dermatological therapeutics *in vivo* using a split-body experimental platform. Prior to initiating such studies, bioluminescence activity was monitored in the hindpaws of *FLG*-*luc2p*^+/−^ animals at 24 hours intervals for 5 consecutive days. While bioluminescence intensity varied day-to-day, relative left/right luciferase activity was remarkably consistent (Fig. 1e and Supplementary Fig. 1f).

### 3.2 Real-time monitoring of siRNA potency, efficacy and longevity *in vivo*

**Fig. 2:**
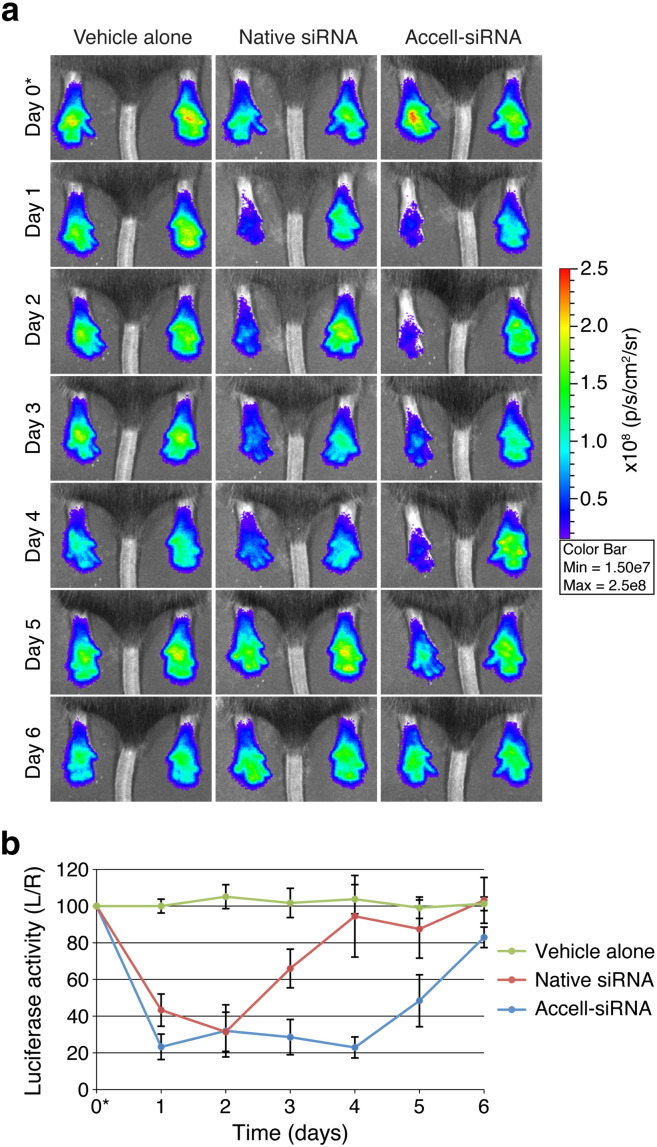
Intradermal injection of unmodified and "self-delivery" siRNAs mediate *in vivo* inhibition of Luc2p activity. (a) *FLG-luc2p*^+/−^ mouse hindpaws (6 animals/group) were intradermally injected with PBS, native-siRNAs, or self-delivery modified Accell™-siRNAs on Day 0 (noted with *). Left paws were treated with siLUC2P-2 (300 pmol) and right paws with NSC4 (300 pmol). 40 µl PBS was injected into both paws of PBS control group. Representative images are shown here (see Supplementary Fig. 2, 3 and 4 for full dataset). (b) Graph depicts the average %L/R ratio for each cohort over the 7-day time-course and the error bars represent standard deviation of the mean.

The hindpaws of *FLG*-*luc2p*^+/−^ mice (n = 6 animals) were injected intradermally with native *luc2p*-targeting (siLUC2P-2; left paw) and non-targeting control (NSC4; right paw) siRNAs. Control animal cohorts (n = 6 animals) were injected with sterile PBS. *In vivo* imaging comparing the left and right hindpaws revealed a 57% inhibition of luciferase activity 24 h after native-siLUC2P-2 siRNA treatment (Fig. 2, Day 1 and Supplementary Figs. 2 and 3, Day 1). Maximum inhibition (69%) was recorded 48 h post-injection (Day 2), after which luciferase activity rapidly returned to baseline within another 48 h (Fig. 2, Day 4). Compared to control cohorts, which showed no appreciable change in left/right luciferase activity, each animal treated with native-siLUC2P-2 siRNA displayed dramatic luciferase activity inhibitions (Supplementary Figs. 2 and 3; p ≤ 10^− 6^ at Days 1 and 2).

Accell™ “self-delivery” modified siRNAs (Accell™-siRNA) facilitate transfection reagent-free *in vitro* gene silencing in keratinocyte monolayers (Supplementary Fig. 4) and organotypic skin models [24]. Here, we compared the *in vivo* potency and longevity of Accell™-siRNAs relative to native-siRNAs in *FLG*-*luc2p*^+/−^ mice. Intradermal injection of Accell™-siLUC2P-2 siRNAs (n = 6 animals) produced more efficient and sustainable *luc2p* knockdown *in vivo* (Fig. 2 and Supplementary Fig. 5). After only 24 h, luciferase was reduced by 77% (Fig. 2, Day 1). Moreover, while native-siRNAs produced similar knockdown effects for the first 48 h (up to Day 2), Accell™-siRNAs mediated knockdown effects were sustained 48 h longer (up to Day 4) and returned to baseline more slowly compared to native siRNA treatment (Fig. 2b).

### 3.3 Comparative analysis of commercial *in vivo* transfection reagents

**Fig. 3:**
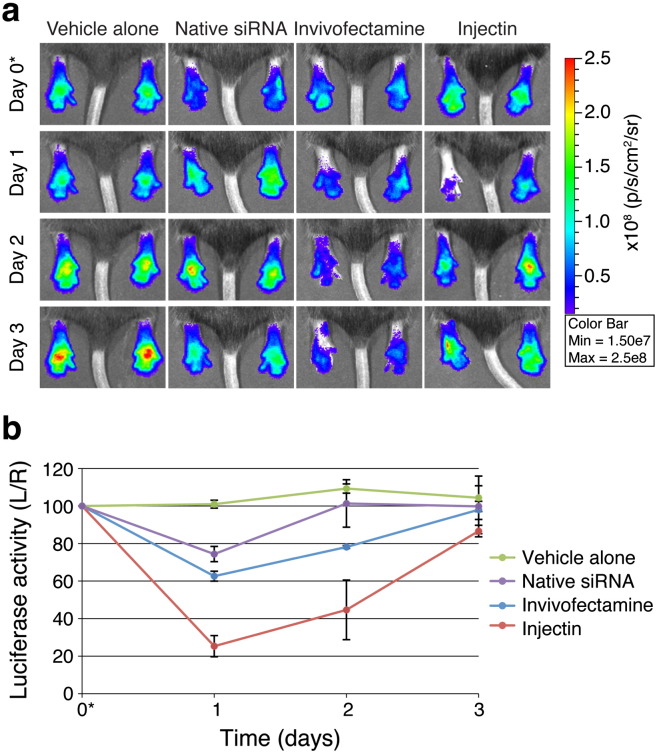
Injectin augments native siRNA-mediated luciferase inhibition *in vivo*. (a) Hindpaws of *FLG-luc2p*^+/−^ mice (3 animals/group) were intradermally injected with PBS, native siRNAs, Invivofectamine^®^ 2.0-complexed native-siRNAs or Injectin-complexed native-siRNAs on Day 0 (noted with *). Left paws were treated with siLUC2P-2 (60 pmol) and right paws with NSC4 (60 pmol). 40 µl PBS was injected into both paws of PBS control group. Representative images are shown here (see Supplementary Fig. 6 for full dataset). (b) Graph depicts the average %L/R ratio for each cohort over the time-course and the error bars represent standard deviation of the mean.

The ability of the lipid-based *in vivo* transfection reagents Invivofectamine® 2.0 or Injectin to enhance native-siRNA silencing *in vivo* was evaluated. Preliminary intradermal injection studies suggested that larger amounts of Invivofectamine® 2.0 were toxic when administered this way (data not shown). Therefore, the amount of native-siRNA used for these studies was reduced to 60 pmol/injection to minimize the amount of transfection reagent injected. The hindpaws of *FLG-luc2p*^+/−^ mice (n = 3 animals/group) were intradermally injected with complexed (Injectin- or Invivofectamine® 2.0) or uncomplexed native-siLUC2P-2 (left paw) and NSC4 (right paw) siRNAs. Control animal cohorts were injected with PBS. While injection of uncomplexed native-siLUC2P-2 produced modest 26% luciferase inhibition 24 h post-injection (Day 1), coupling the same amount of native-siRNA with Injectin resulted in a dramatic 75% inhibition (Fig. 3 and Supplementary Fig. 6). Unfortunately, these knockdown effects were not sustained, and luciferase activities quickly returned to baseline within 72 h (Day 3). Injection of even small amounts of Invivofectamine® 2.0 resulted in edema of the paw, possibly masking the *in vivo* activity of the siRNA as only 37% luciferase inhibition was observed (Fig. 3 and Supplementary Fig. 6).

### 3.4 Testing alternative gene silencing reagents *in vivo*

**Fig. 4:**
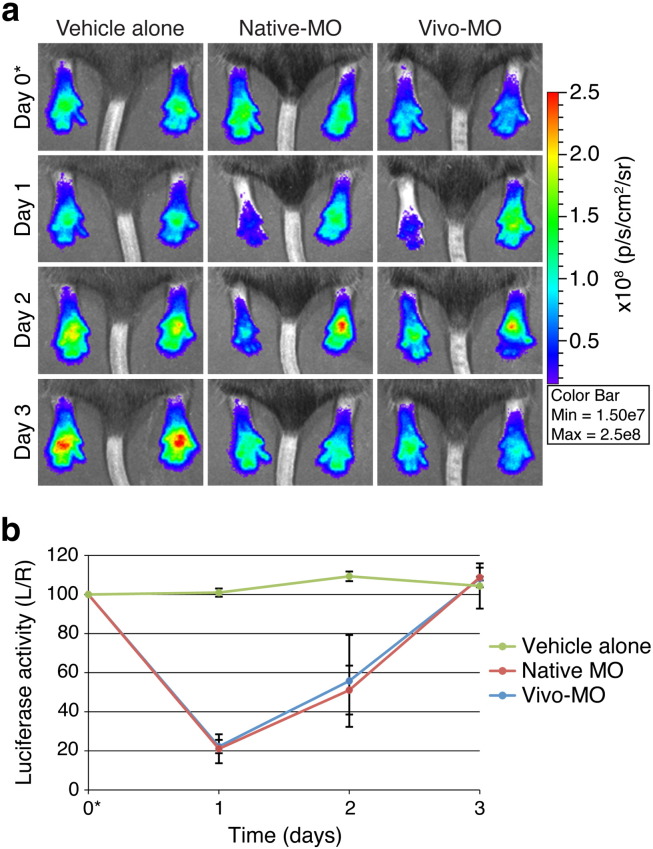
Morpholino antisense oligonucleotides inhibit luciferase activity *in vivo*. (a) Paws of *FLG-luc2p*^+/−^ mice (3 animals/group) were intradermally injected with PBS, native morpholino antisense oligonucleotides (native-MO), or self-delivery modified MOs (Vivo-MO) on Day 0 (noted with *). Left paws were treated with LUC2P-MO (300 pmol) and right paws with control-MO (300 pmol). 40 µl PBS was injected into both paws of PBS control group. Representative images are shown here (see Supplementary Fig. 7 for full dataset). (b) Graph depicts the average %L/R ratio for each treatment group over the 7-day time-course and the error bars represent standard deviation of the mean.

To explore the *in vivo* efficacy of alternative nucleic acid gene silencing reagents, native morpholino and “self-delivery” modified Vivo morpholino antisense oligonucleotides were evaluated in *FLG-luc2p*^+/−^ mice (n = 3 animals/group). Hindpaws were injected with native or Vivo-modified *luc2p*-targeting MO (LUC2P-MO; left paw) and control-MO (right paw), while control animal cohorts were injected with PBS alone. *In vivo* imaging revealed that native- and Vivo-MOs produced similar knockdown effects 24 h post-treatment (Fig. 4, Day 1 and Supplementary Fig. 8). Unfortunately, these effects were short-lived and luciferase signals returned to baseline within 72 h (Day 3).

### 3.5 Non-invasive, topical delivery of siRNA into the epidermis

As mentioned above, a painless (albeit non- or minimally-invasive) delivery system is required for clinical application of siRNA-based therapies within dermatology. A recent study demonstrated that topical application of siRNA-based spherical nucleic acid gold nanoparticles mixed in an over-the-counter dermatological ointment (Aquaphor®) conferred potent *in vivo* target inhibition in mouse back skin [19]. This prompted us to formulate our *luc2p*-targeting siRNAs in Aquaphor® and test their delivery into the paw skin of *FLG-luc2p*^+/−^ mice. For five consecutive days, native- or Accell™-siRNAs were mixed with Aquaphor® healing ointment and applied to the hindpaws of anesthetized mice (n = 3 animals/group) for 45 min/day. Luciferase activity was monitored *via in vivo* imaging prior to initial Aquaphor®/siRNA application (Supplementary Fig. 8, Day 0) and at 24 hour intervals for 6-days thereafter (Supplementary Fig. 8, Days 1–6). Aquaphor® alone did not alter luciferase activity. Encouragingly, both native- and Accell™-siLUC2P-2 reduced luciferase activity by 17% and 20%, respectively, within 48 h of the first treatment (Supplementary Fig. 8, Day 2). This level of inhibition was maintained during treatment and for 24 h (Day 5) after treatment termination.

As mentioned above, a painless (albeit non- or minimally-invasive) delivery system is required for clinical application of siRNA-based therapies within dermatology. A recent study demonstrated that topical application of siRNA-based spherical nucleic acid gold nanoparticles mixed in an over-the-counter dermatological ointment (Aquaphor®) conferred potent *in vivo* target inhibition in mouse back skin [19]. This prompted us to formulate our *luc2p*-targeting siRNAs in Aquaphor® and test their delivery into the paw skin of *FLG-luc2p*^+/−^ mice. For five consecutive days, native- or Accell™-siRNAs were mixed with Aquaphor® healing ointment and applied to the hindpaws of anesthetized mice (n = 3 animals/group) for 45 min/day. Luciferase activity was monitored via *in vivo* imaging prior to initial Aquaphor®/siRNA application (Supplementary Fig. 8, Day 0) and at 24 hour intervals for 6-days thereafter (Supplementary Fig. 8, Days 1–6). Aquaphor® alone did not alter luciferase activity. Encouragingly, both native- and Accell™-siLUC2P-2 reduced luciferase activity by 17% and 20%, respectively, within 48 h of the first treatment (Supplementary Fig. 8, Day 2). This level of inhibition was maintained during treatment and for 24 h (Day 5) after treatment termination.

**Fig. 5:**
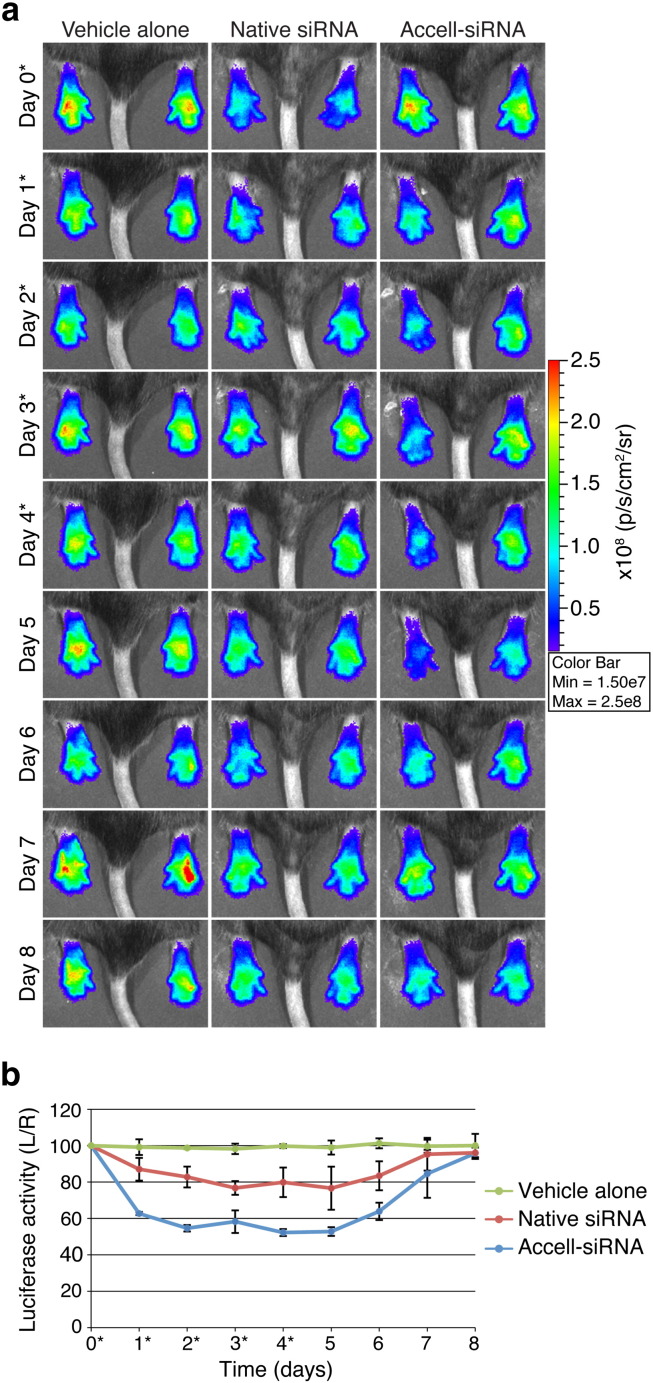
50% *in vivo* inhibition of luciferase activity achieved via topical delivery of Accell^™^-siRNAs using a novel siRNA-ointment formulation. (a) 40 µg of the Aquaphor^®^-PG-siRNA (native or Accell^™^) was applied to the paws of *FLG-luc2p*^+/−^ mice (3/group) for 50 min; treatments were repeated every 24 h for 5 days (Days 0-4; noted with *). Left paws were treated with siLUC2P-2 (300 pmol) and right paws with NSC4 (300 pmol). Control group received the Aquaphor^®^-PG without siRNA on the left paw and no treatment on the right paw. Representative images are shown here (see Supplementary Fig. 9 for full dataset). (b) Graph depicts the average %L/R ratio for each treatment group over the 9-day time-course.

Chemical penetration enhancers, like propylene glycol (PG) that reversibly alter the barrier properties of the *stratum corneum* and potentially increase drug solubility [36,37], are often used in dermatology to enhance topical drug delivery. Based on this, and the encouraging results obtained with Aquaphor® alone, we developed a novel Aquaphor®-based topical formulation containing propylene glycol (PG) and determined whether enhanced delivery and subsequent *in vivo luc2p* knockdown could be achieved. Native- or Accell™-siRNAs were mixed with an Aquaphor® ointment containing PG (20% w/v) and applied to the hindpaws of *FLG-luc2p*^+/−^ mice as described above (n = 3 animals/group) and luciferase activity was monitored for 9-days (Fig. 5; Supplementary Fig. 9). Addition of PG failed to significantly enhance native-siLUC2P-2 mediated luciferase inhibition. Remarkably, however, addition of PG resulted in 37% *luc2p* knockdown after the first application of Accell™-siLUC2P-2. Accell™-mediated silencing peaked at 48% on Day 2 48 h after initial application. This level of knockdown was sustained as treatment continued and for an additional 24 h (through Day 5) following the final treatment (Fig. 5). Luciferase activity then slowly returned to baseline. Similar results were obtained with native- or Accell™-siLUC2P-2 and an unrelated non-pathway targeting native- or Accell™ control siRNA (siNTP; n = 3 animals/group; data not shown).

## 4 Discussion

RNAi-based therapeutics show compelling potential for treating various skin conditions [9–12], specifically the dominant-negative subset, which includes numerous keratin disorders [2,3,38]. siRNAs that specifically target mutant keratin genes have been designed and tested *in vitro* and *in vivo* in animal models [14,15] and a phase 1b clinical trial [17]. Unfortunately, the pain associated with the intradermal injections used to deliver these siRNAs was intolerable; thus, alternative delivery methods (e.g. topical formulations) are needed. Several studies have described successful topical delivery of siRNAs into the skin [12,18,19], but the lack of a tractable, real-time *in vivo* monitoring system has slowed validation and possible refinement of these delivery approaches. Reporter plasmid/siRNA co-injection studies have demonstrated the efficacies of siRNA inhibitors *in vivo*, but these studies are prone to high variability and in some cases large animal cohorts were required to reach statistical significance [14–16]. Here, we developed an innovative *in vivo* methodology for evaluating epidermal nucleic acid delivery in live animals in real-time. Combining our reporter mouse model (*FLG*-*luc2p*^+/−^) with live animal bioluminescence imaging techniques produces visual, real-time reporter gene activity readouts. Unlike the methodologies previously reported, this workflow allows the user to instantly define experimental outcomes and reduces the need for time-consuming post-treatment validation studies (e.g. qRT-PCR and Western blotting). Moreover, the ability to monitor gene expression in the same mouse everyday greatly reduces the number of animals required for each experiment.

As a proof-of-concept, we used this workflow to compare and contrast the efficacies of different siRNA chemistries, morpholino antisense chemistries and *in vivo* transfection reagents following intradermal injection. While previous studies have looked at the individual efficacies of these reagents *in vitro* and *in vivo*, this methodology has allowed us to perform the first comprehensive, multiple time-point study directly comparing the *in vivo* knockdown capacities of different post-transcriptional gene regulation techniques. Our studies revealed equivalent initial *in vivo* inhibitions for all siRNA and morpholino chemistries evaluated. We found that Invivofectamine® 2.0 caused severe edema when delivered *via* intradermal injection. Injectin, on the other hand, showed no signs of toxicity and required 5-fold less native-siRNA to achieve similar levels of luciferase inhibition as those observed with native- or Accell™-siRNAs alone. Nevertheless, the duration of Accell™-siRNA mediated inhibition was considerably longer compared to all other molecules. Together, these findings indicate that the “self-delivery” and/or backbone stability modifications [26–28] present in Accell™-siRNA prolong target knockdown *in vivo* and identify this siRNA chemistry as an attractive RNAi-based therapeutic for clinical use within dermatology.

As mentioned above, developing an effective, non-invasive epidermal delivery system would represent a key advance towards clinical acceptance and administration of nucleic acid-based therapeutics. Several research groups have made great strides towards developing such delivery methodologies [12,18,19], but the lack of a reliable, real-time *in vivo* monitoring system has slowed topical delivery evaluation and refinement. Using the parameter defined by Zheng et al. [19] as a baseline, we formulated Aquaphor®-siRNA ointments containing native- or Accell™-siRNAs and evaluated their efficacy in our *FLG*-*luc2p*^+/−^ mouse model. Within 48 h of application, modest 20% *luc2p* knockdown was observed. As some researchers suggest that only a 50% reduction in mutant protein levels may be required to produce therapeutic effects for some genetic skin disorders [15], we explored whether the addition of a chemical penetration enhancer, propylene glycol (PG), would improve *in vivo* silencing. Remarkably, topical application of Accell™-siRNAs formulated in this novel Aquaphor®/PG ointment resulted in a 2-fold increase in luciferase inhibition compared to Aquaphor® alone. Indeed, ~ 50% reporter inhibition was achieved, which suggests that this new topical formulation may be a truly viable candidate for siRNA-based therapeutics for skin disorders. Importantly, previous animal model studies have demonstrated that a 1MUT:2WT *in vivo* allele expression ratio results in morphologically normal and functional skin [39]. These findings encouragingly suggest that the ~ 50% reduction in target gene expression that we achieved via topical siRNA delivery may be sufficient to treat human keratinizing skin disorders.

Linking the expression of luciferase reporter gene to the human filaggrin promoter has given this reporter mouse model added value within the field of investigative dermatology. *FLG* encodes profilaggrin, a precursor protein that is post-translationally processed into filaggrin monomers, which help form the skin barrier and are vital for the health and appearance of the skin [5,40]. *FLG* loss-of-function mutations are predisposing factors for the very common human skin conditions icthyosis vulgaris and atopic dermatitis (eczema) [41–43]. It has, therefore, been suggested that up-regulating *FLG* expression may prove therapeutically beneficial for one, or all, of these conditions. While we have not demonstrated alternative uses of the *FLG*-*luc2p*^+/−^ animal model in the current study, it represents the ideal *in vivo* experimental platform for testing the efficacy of compounds that regulate *FLG* gene expression.

## 5 Conclusions

The work presented here describes a unique and reliable reporter mouse model that is ideally suited for rapid and robust real-time *in vivo* evaluation of novel therapeutics within dermatology. We have developed a work-flow that has allowed us to compare and contrast the *in vivo* efficacies of various nucleic acid-based therapeutic in real-time. Importantly, this real-time monitoring work-flow has allowed us to develop a novel topical formulation that non-invasively delivers both uncomplexed native-siRNAs and “self-delivery” modified Accell™-siRNAs into the epidermis. Remarkably, the effectiveness of this relatively simple, clinic-ready topical formulation suggests that non-invasive therapeutic delivery of siRNA into the skin may not be as challenging as previous studies have suggested and brings RNAi-based therapeutics much closer to clinical use.

## Appendix A Supplementary data

Supplementary Information. Includes Supplementary Materials and Methods, Supplementary Table 1 and Supplementary Figures 1-9.
