# Supplementary material for: In vivo gene silencing following non-invasive siRNA delivery into the skin using a novel topical formulation
Source: J Control Release. 2014 Dec 28;196:355–62. doi: 10.1016/j.jconrel.2014.10.022 (PMC4275573; doi:10.1016/j.jconrel.2014.10.022)
Supplement: Supplementary file 1 — Supplementary Information. Includes Supplementary Materials and Methods, Supplementary Table 1 and Supplementary Figures 1-9. [file mmc1.pdf]

Supplementary Materials and Methods

Recombineering of *FLG*-10k human filaggrin promoter fragment

The *FLG*-10k human filaggrin promoter fragment was assembled from bacterial artificial chromosome (BAC) clone RP1-14N1 via a two-step recombineering process. A 10.1 kb fragment containing a 5' *Xho*I restriction site, ~10 kb upstream of the transcription *FLG* start site, exon 1 (partial 5'UTR), the first 18-bp of intron 1, and a 3' *Mlu*I restriction site was amplified from the BAC clone using primers mentioned in Supplementary Table 1. A second, 483-bp fragment containing a 5' *Mlu*I restriction site, the last 459-bp of intron 1, the start of exon 2 encompassing the remainder of the 5'UTR and a 3' *HinD*III restriction site was amplified from the same BAC clone. Fragments were sequence-verified and ligated via their *Mlu*I sites, generating the 10.6 kb hFLG-10k human filaggrin promoter fragment.

Supplementary Table 1: *Cloning primers*

	Sequence
Right arm:<br>Human Genome Build 18 (hg18), chromosome 1	5' – GGT AAG CAA TAT GAA AAC AAT TTG TAG CTC ATT CAC TGC CAG ACA CTG ACT CGA GAC AAC TTA TAT CGT ATG GGG C – 3'
Left arm:<br>Human Genome Build 18 (hg18), chromosome 1	5' – TCC TTC AGG CTA CAT TCT ATT TGC TCT TTT GGT GAA CAA GGT AAG AAG GAA TAC GCG TTA CGC CCC GCC CTG CCA C – 3'
FLG homology *Mlu*I	5' – AGG TAA GTC ACG CGT ATC TTG TCA TAT GGC TAA CTG G – 3'
FLG homology *HinD*III	5' – TCA AGC TTT TGG CAA TAA ATG TGA ACC – 3'

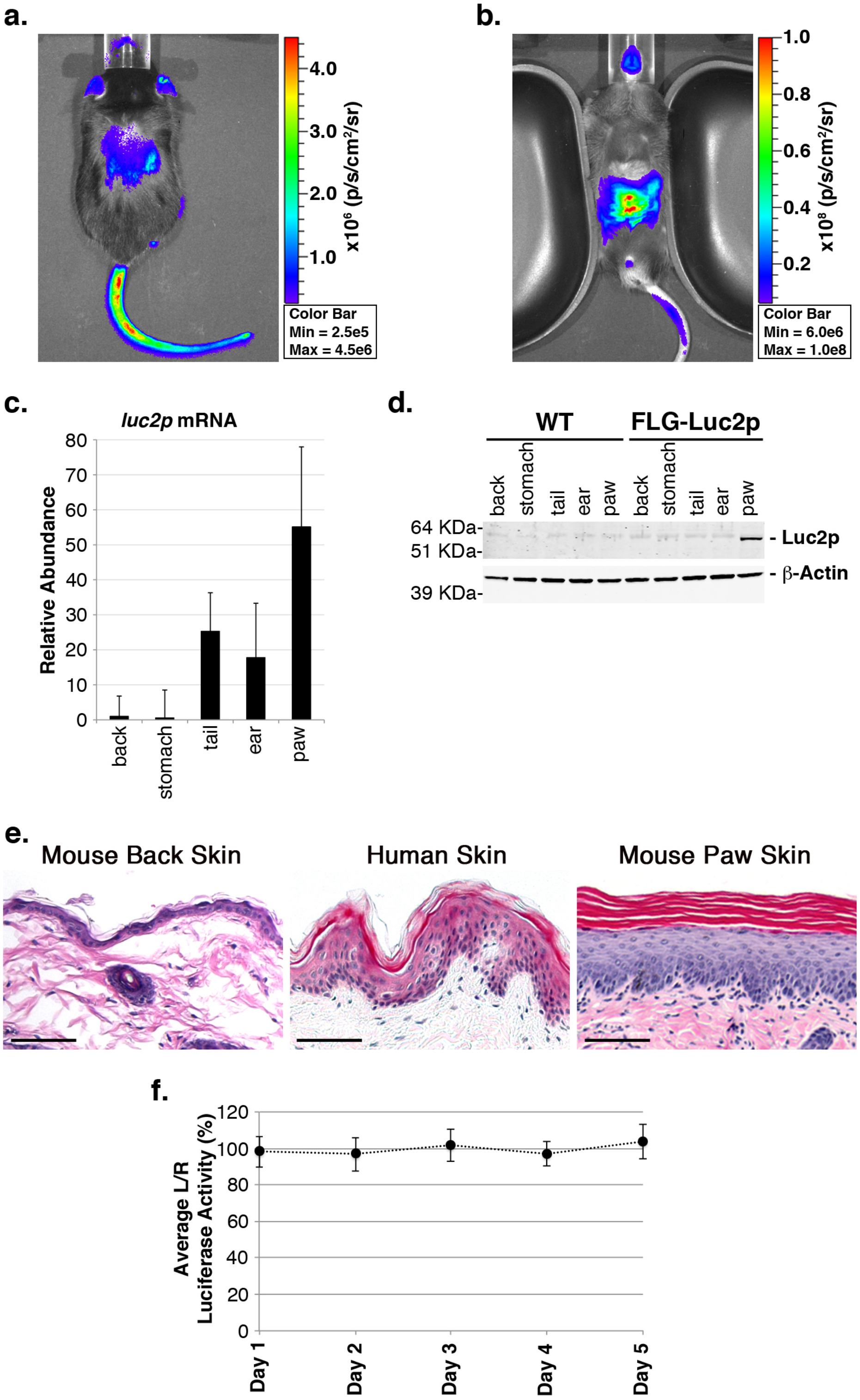

Supplementary Figure 1: *Further characterization of the* FLG-luc2p$^{+/-}$ *mouse model.* (**a-b**) Luciferin was administered to *FLG-luc2p$^{+/-}$* mice via intraperitoneal injection and imaged for 1 min, 10 min post-injection using the IVIS Lumina. Moderate luciferase bioluminescence activity was detected in the tail, shaved back and stomach, ears, perioral and perianal regions under these conditions. (**c**) Taqman-based qRT-PCR analysis demonstrated that luc2p mRNA was more abundant in *FLG-luc2p$^{+/-}$* paw tissues relative to back, stomach, ear and tail epidermal tissues. (**d**) Firefly luciferase protein was only detectable by western blotting in *FLG-luc2p$^{+/-}$* paw tissues. Membranes were simultaneously probed with anti-β-actin antibodies as a loading control. (**e**) H&E-stained cross-sections of wild-type mouse back skin (left panel), human skin (middle panel) and wild-type mouse paw skin (right panel). Mouse back skin generally comprises only three cell layers and has a very thin stratum corneum layer, in contrast both human epidermis and mouse paw epidermis are made up of 6–10 keratinocyte cell layers and a well-defined stratum corneum barrier. Scale Bar = 100 μm. (**f**) Graph depicts the average %L/R ratio (n=12) at each timepoint over a 5-day baseline *in vivo* imaging time-course and the error bars represent standard deviation of the mean.

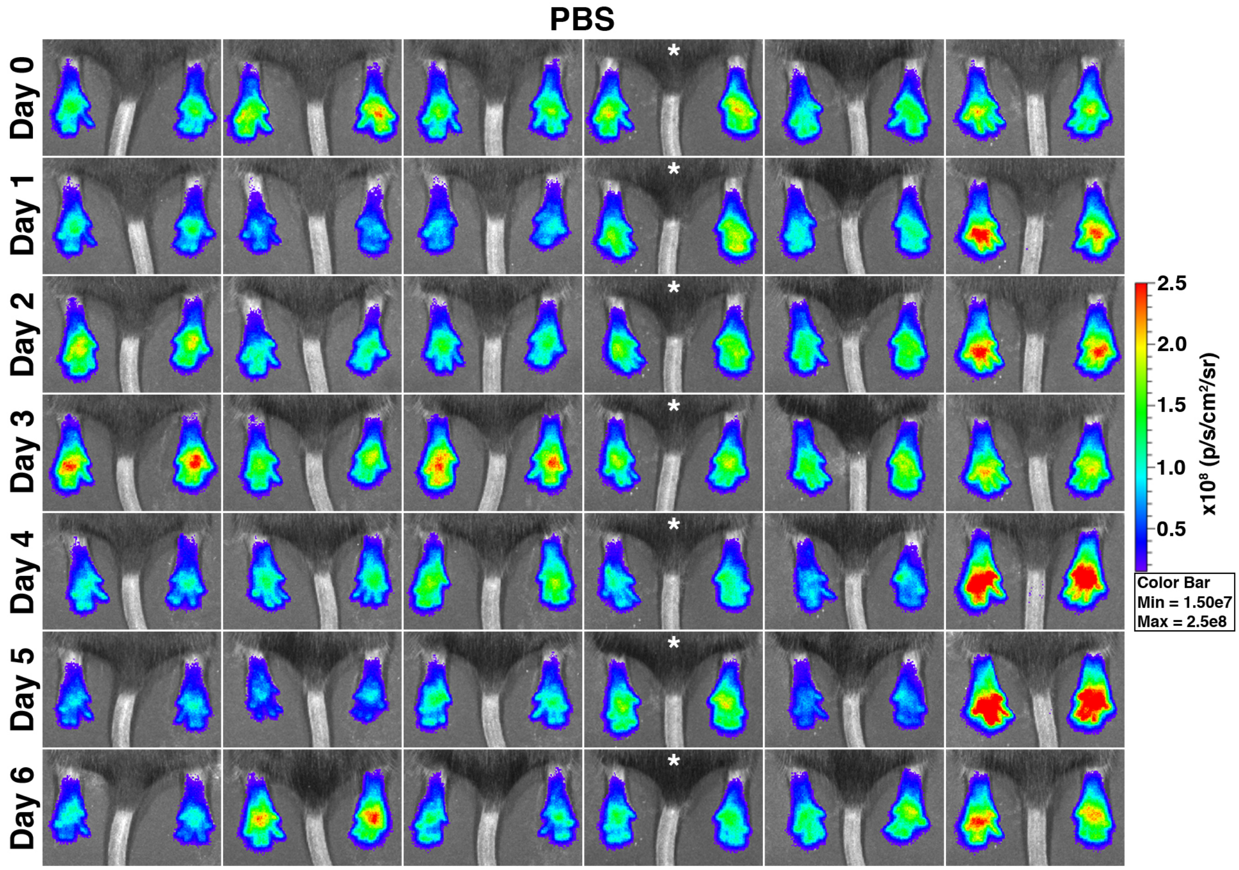

Supplementary Figure 2: *Full experimental dataset for PBS control treatments from native- and Accell-siRNA* in vivo *study presented in Fig. 2. In vivo* images depicting the LLEs of all PBS control group animals from the intradermal injection native- and Accell-siRNA study. Images denoted by * represent the subject presented in **Fig. 2a**.

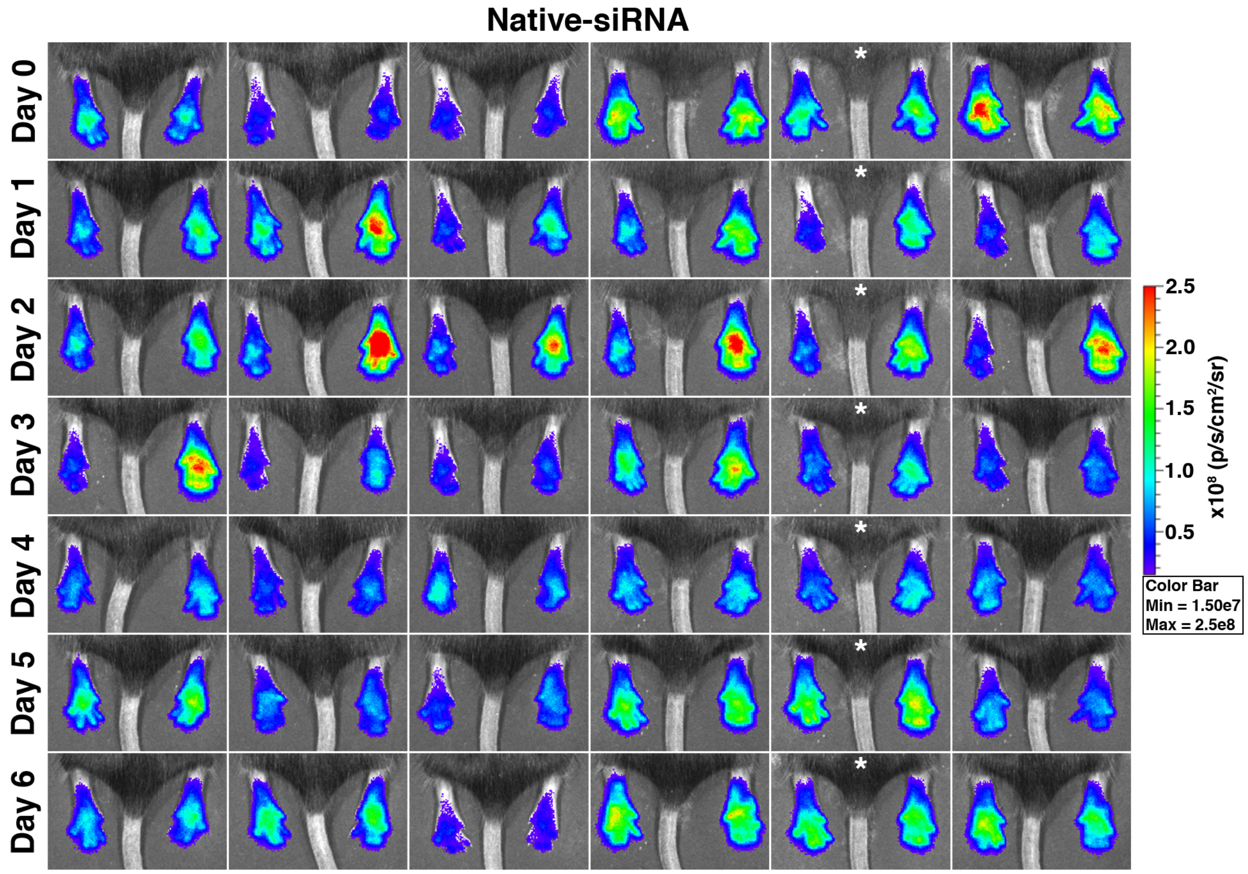

Supplementary Figure 3: *Full experimental dataset for native-siRNA treatments from native- and Accell-siRNA* in vivo *study presented in Fig. 2. In vivo* images depicting the LLEs of all native-siRNA treated animals from the intradermal injection native- and Accell-siRNA study. Images denoted by * represent the subject presented in **Fig. 2a**.

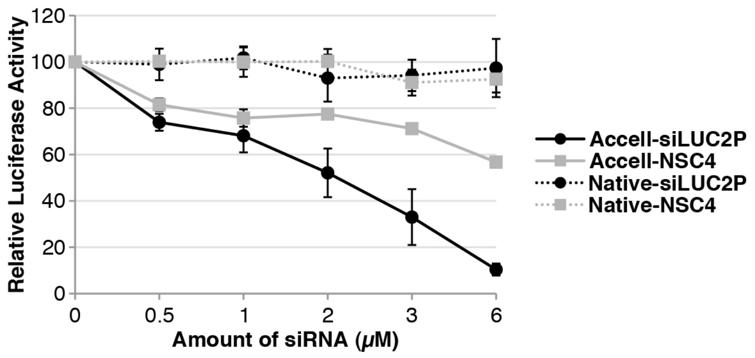

Supplementary Figure 4: In vitro *validation of self-delivery siRNA*. *pK6a-Luc2p*-HACAT cells were treated with increasing concentrations of native or self-delivery modified siLUC2P-2 or NSC4 siRNAs. Cell viability assays (resazurin metabolism) and firefly luciferase activities were measured 48 hours after treatment. Resazurin normalized firefly luciferase activities are expressed as percentages of activity at 0 μM. Error bars indicate standard deviations of the mean for biological replicate experiments (n = 3). Native-siRNAs had no knockdown effect on Luc2p activities *in vitro*. In contrast, Accell-siLUC2P-2 siRNAs specifically inhibited Luc2p activity at concentrations >2 μM.

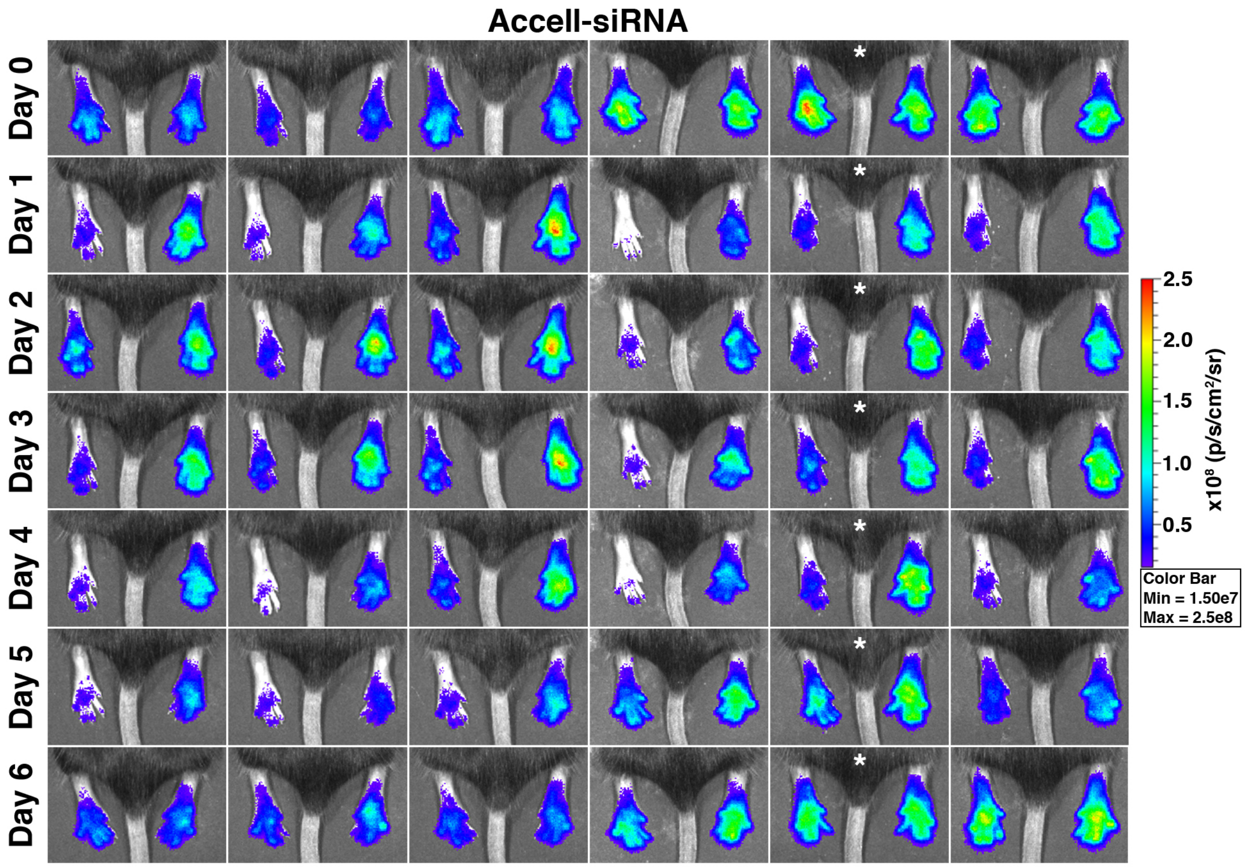

Supplementary Figure 5: *Full experimental dataset for Accell-siRNA treatments from native- and Accell-siRNA* in vivo *study presented in Fig. 2. In vivo* images depicting the LLEs of all Accell-siRNA treated animals from the intradermal injection native- and Accell-siRNA study. Images denoted by * represent the subject presented in **Fig. 2a.**

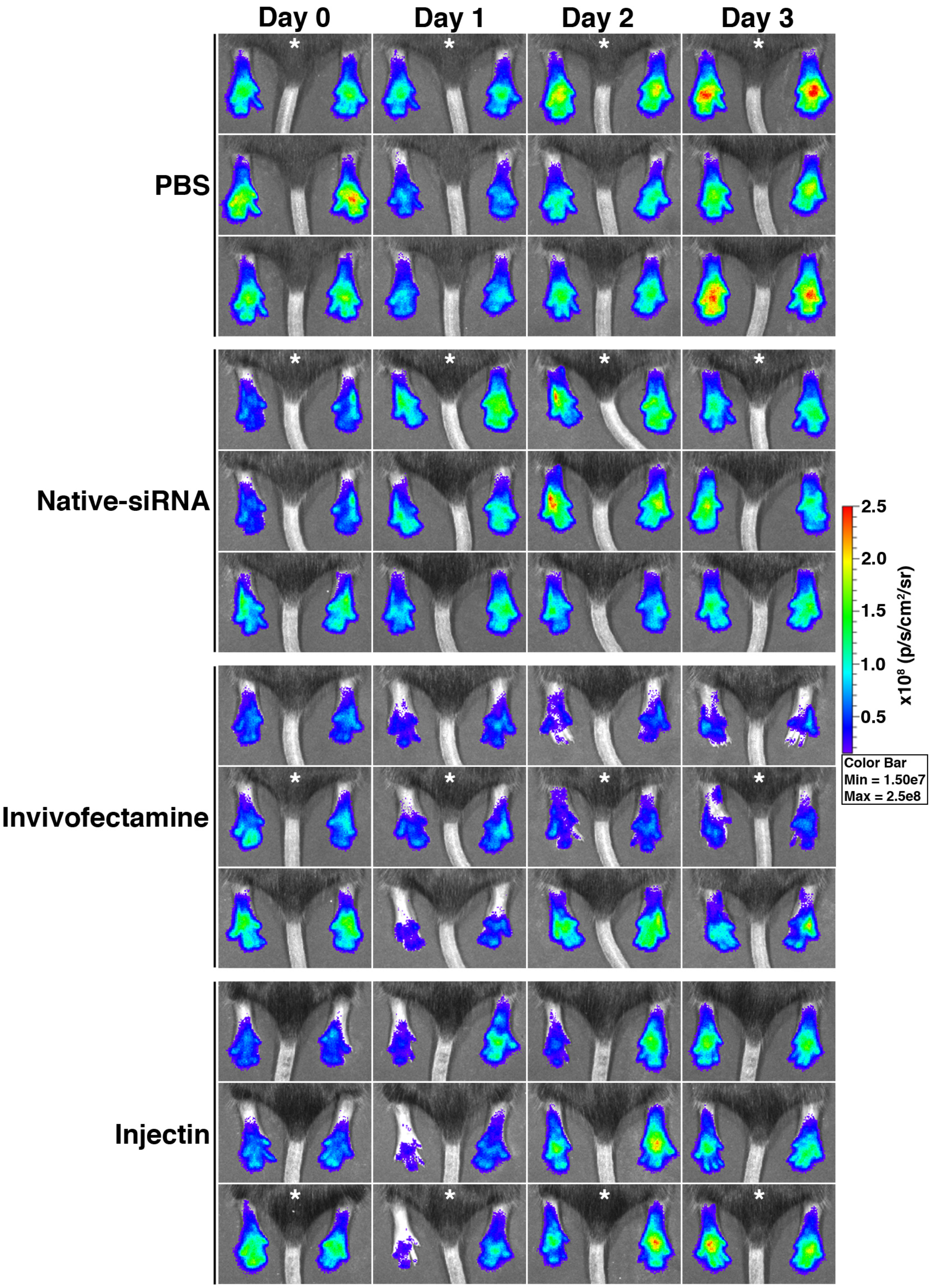

Supplementary Figure 6: *Full experimental dataset for native-siRNA, Invivofectamine 2.0 and Injectin intradermal injection* in vivo *study presented in Fig. 3. In vivo* images depicting the LLEs of all animals intradermally injected with PBS, native-siRNA, Invivofectamine 2.0-complexed native-siRNA or Injectin-complexed native-siRNA. Images denoted by * represent the subject presented in **Fig. 3a**.

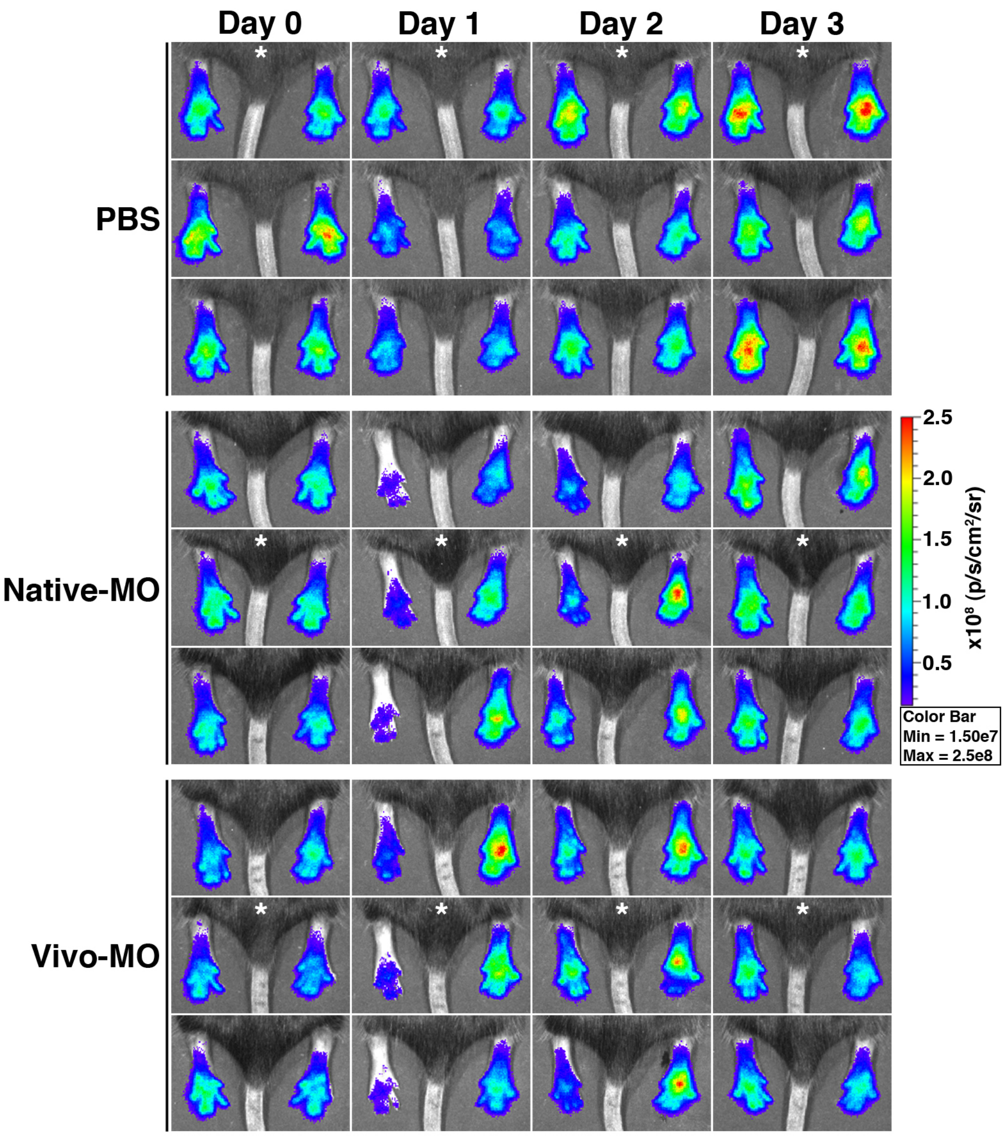

Supplementary Figure 7: *Full experimental dataset for native and self-delivery modified MO* in vivo *study presented in Fig. 4. In vivo* images depicting the LLEs of all animals intradermally injected with PBS, native-MOs, or Vivo-MO. Images denoted by * represent the subject presented in **Fig. 4a**.

a.

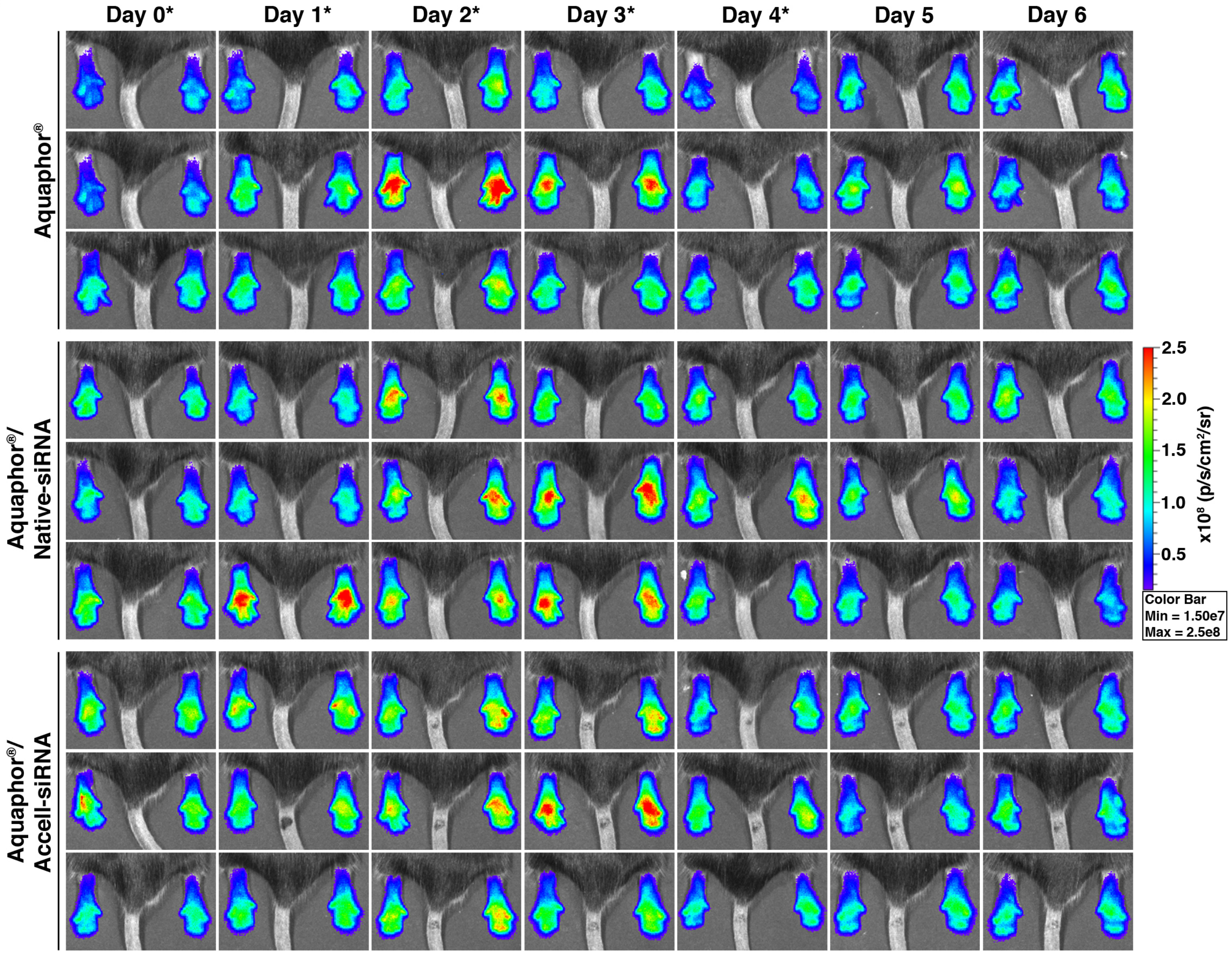

b.

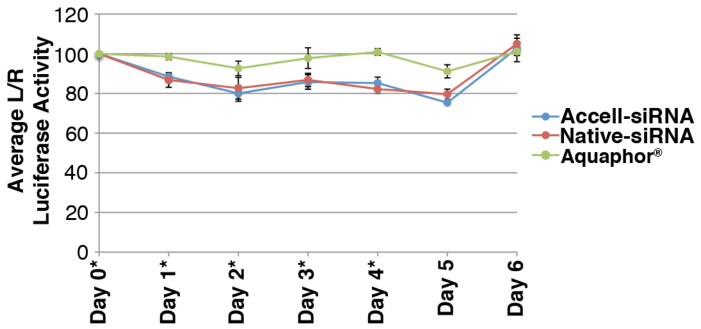

Supplementary Figure 8: *Inhibition of luciferase activity achieved via topical delivery of siRNAs using Aquaphor® Healing Ointment.* (**a**) Aquaphor® Healing Ointment containing native- or Accell-siRNAs was topically applied to the footpads of anesthetized *FLG-luc2p*$^{+/-}$ mice (3/group) for 50 min; treatments were repeated every 24 h for 5 days (Days 0-4). Left paws were treated with siLUC2P-2 (300 pmol) and right paws with NSC4 (300 pmol). Control group received Aquaphor® ointment on the left paw and no treatment on the right paw. Luciferase activity was monitored at 24 h intervals until signals returned to baseline (%L/R ratio ≈ 100; Day 6). (**b**) Graph depicts the average %L/R ratio for each treatment group over the 7-day time-course and the error bars represent standard deviation of the mean.

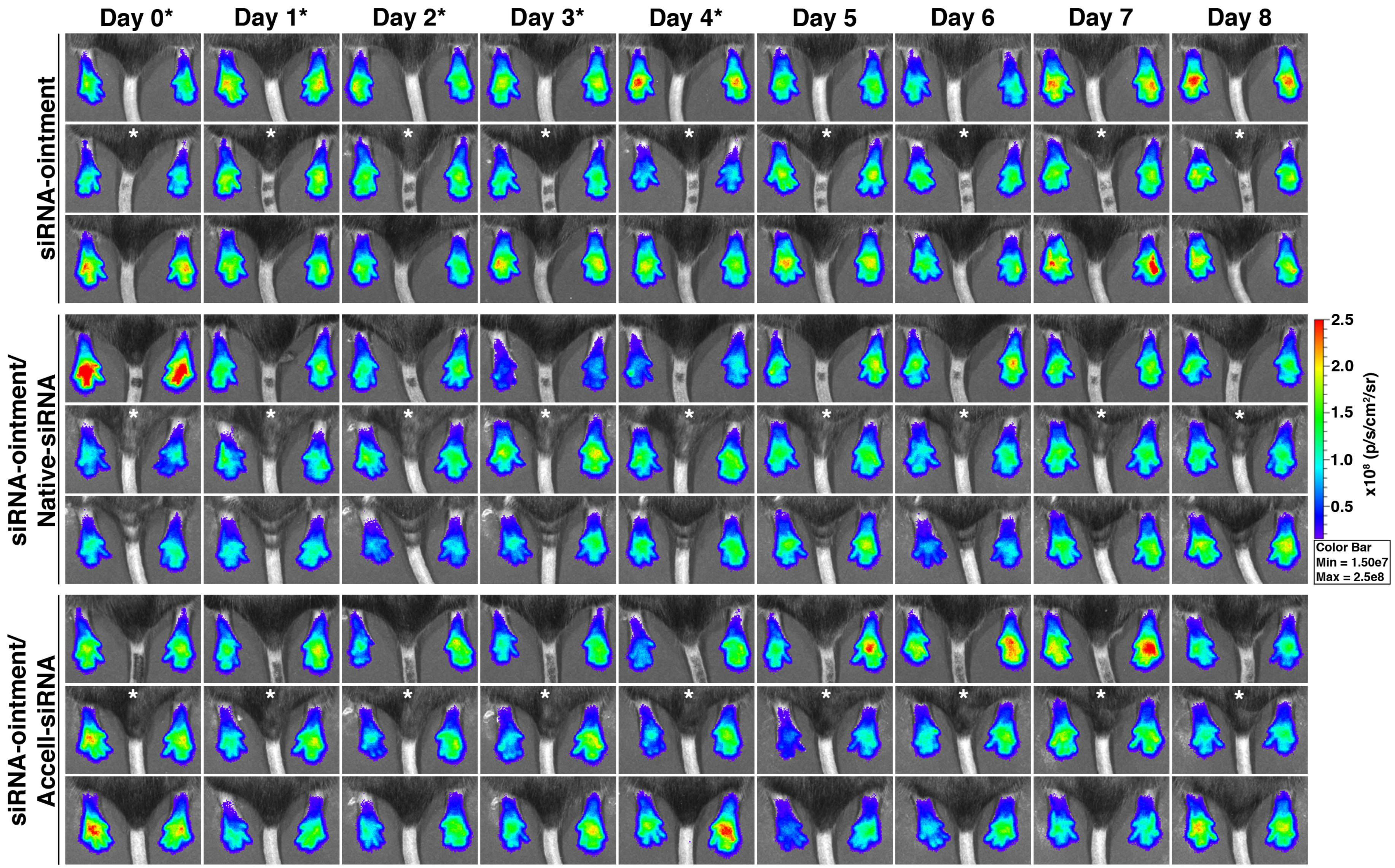

Supplementary Figure 9: *Full experimental dataset for the siRNA-ointment formulation* in vivo *study presented in Fig. 5. In vivo* images depicting the LLEs of all animals treated with the Aquaphor®-PG alone or with 300 pmol native- or Accell-modified siRNAs. Images denoted by * represent the subject presented in **Fig. 5a.**
